# Supplementary material for: Personal attitude or experience? Which factors influence residents’ acceptance of mixed-income communities?
Source: PLoS One. 2021 Apr 23;16(4):e0250511. doi: 10.1371/journal.pone.0250511 (PMC8064591; doi:10.1371/journal.pone.0250511)
Supplement: S1 Appendix — (DOCX) [file pone.0250511.s001.docx]

**S1 Appendix**

**The survey on residents’ perception
 and acceptance towards shared housing and mixed-income communities**

**Dear Sir/Madam,**

This survey belongs to the project funded by The Ministry of Land, Infrastructure and Transport's R&D titled: *“Development of shared housing model based on residents’ behavior analysis*.*”*

The survey aims to investigate the perception and acceptance of the general residents on social mix for scientific purposes. Therefore, the information of your response is protected as personal confidentiality in this investigation in accordance with Article 33 of the Statistics Act (Protection of Secrets).

In this way, we appreciate your precious time to attend to the survey. Your response will provide valuable data for the development of shared housing models and policy proposals.

**Article 33 (Protection of Secrets)** (1) Matters belonging to the confidential information of individuals, corporations, organizations, etc. that have become known in the course of producing statistics shall be protected.

(2) Data belonging to the confidential information of individuals, corporations, organizations, etc. that have been produced for the production of statistics shall not be used for any purpose other than that of producing statistics.

**Article 34 (Duty of Statistics Staff, etc.)** Any person who is or was a statistics staff member or who is or was engaged in the business of producing statistics after being entrusted with all or part of such business shall not use the information that they have come to know in the course of conducting the duty for any purpose other than the relevant duty, nor shall provide them to other persons.

**A. SELECTION CHOICE (for sampling)**

*Please answer these questions:*

S1. What is your gender?

1. Male ② Female

S2: How old are you?

Age ( )

S_3. Which city do you live in?

1. Seoul
2. Busan
3. Daegu
4. Incheon
5. Gwangju
6. Daejeon
7. Ulsan

S_3-1. If you live in Seoul, which living zone do you live in?

1. Central
2. NE
3. NW
4. SW
5. SE

S.4 Please specify your current address up to Dong level.

( ) City/Do ( ) District/Gun/Gu
( ) Ward/Eup/Myeon/Dong

**A. GERNERAL INFORMATION**

A1. Which of the following is your current marital status?

1. Single
2. Married
3. Divorced
4. Widowed

A2. How many people do you live with at your current house? ( )

A3. How many children under 19 years old do you have? ( )

A4. Which type of house do you reside in?

1. Apartment
2. Efficiency apartment (Officetel)
3. Single-family house
4. Multifamily house
5. Studio
6. A slice room (JJokbang/Gosiwon)
7. Houses in non-residential buildings (commercial buildings, factories)
8. Other. Please specify: ( )

A5. What is your house tenure type?

1. Owner
2. Chonse
3. Monthly payment with deposit
4. Monthly payment without deposit
5. Sakwolse or annual payment
6. Daily rent
7. Free of rent

*Chonse: A renter makes a lump sum deposit of “key money” at the beginning of occupancy which is fully refunded at the end of the contract period. The landlord usually invests this money and interest earning it represents in imputed rent. (Source: Ha, S. K. (2002). The urban poor, rental accommodations, and housing policy in Korea. *Cities, 19(3)*, 195-203.).

*Sakwolse: This is a lump sum payment of rent for six months or a year at the beginning of the rent occupancy.

A6. Please check the income group you believe you are included in:

1. Lower-income
2. Middle income
3. Higher-income

A7. What is your final educational attainment? If you are dropping out, please answer based on the academic background you graduated from.

1. Under high school graduation
2. High school graduation
3. Currently enrolled in undergraduate college/university
4. Undergraduate graduation (University graduation)
5. Currently enrolled in graduate school
6. Graduate school graduation (masters / Ph.D. / doctorate)

A8. What is your average monthly gross household income? ( ) (unit: 10,000 KRW)

**B. PERSONAL ATTITUDE AND EXPERIENCE OF SOCIAL DIVERSITY**

B1. Please check the political propensity tendency you believe you are included in

| Conservative | Moderately conservative | Neutral | Moderately progressive | Progressive |
| --- | --- | --- | --- | --- |
| ① | ② | ③ | ④ | ⑤ |

B2. How do you consider a neighborhood you currently live in?

| **No.** | **Items** | **Strongly disagree** | **Disagree** | **Agree** | **Strongly agree** |
| --- | --- | --- | --- | --- | --- |
| 1 | My neighborhood is a great place to live while doing what I want to do. | ➀ | ➁ | ➂ | ➃ |
| 2 | I will continue to live in my neighborhood if it is possible. | ➀ | ➁ | ➂ | ➃ |
| 3 | I am proud to live in my neighborhood. | ➀ | ➁ | ➂ | ➃ |
| 4 | I have a neighbor who cares about me. | ➀ | ➁ | ➂ | ➃ |
| 5 | I can actively help my neighbors in my neighborhood if they are in troubles. | ➀ | ➁ | ➂ | ➃ |
| 6 | I can find someone to talk to in my neighborhood if I need to. | ➀ | ➁ | ➂ | ➃ |
| 7 | I greet when I meet neighbors on the street in my neighborhood. | ➀ | ➁ | ➂ | ➃ |
| 8 | I would like to contribute to the development of my neighborhood. | ➀ | ➁ | ➂ | ➃ |
| 9 | I feel that I belong to my neighborhood | ➀ | ➁ | ➂ | ➃ |
| 10 | I am willing to actively participate in a project of my neighborhood. | ➀ | ➁ | ➂ | ➃ |
| 11 | I am happy to live in my neighborhood. | ➀ | ➁ | ➂ | ➃ |

B3. Please check your opinion about the below statements.

| **No.** | **Items** | **Strongly disagree** | **Disagree** | **Agree** | **Strongly agree** |
| --- | --- | --- | --- | --- | --- |
| 1 | I can be a family member of people who have different socioeconomic backgrounds. | ➀ | ➁ | ➂ | ➃ |
| 2 | I can be a friend of people who have different socioeconomic backgrounds. | ➀ | ➁ | ➂ | ➃ |
| 3 | I can be a neighbor of people who have different socioeconomic backgrounds. | ➀ | ➁ | ➂ | ➃ |
| 4 | I would like to accept people having different socioeconomic backgrounds from me to live in my neighborhood. | ➀ | ➁ | ➂ | ➃ |
| 5 | I am willing to accept to live in a neighborhood where people having different socioeconomic backgrounds from me, reside. | ➀ | ➁ | ➂ | ➃ |

B4. How many times do you meet with below groups?

| **Relationships** | **Income levels** | **None** | **Once a year** | **Once in six months** | **Once per month** | **Once per week** | **Almost every day** |
| --- | --- | --- | --- | --- | --- | --- | --- |
| Family/ relatives | Lower-income | ➀ | ➁ | ➂ | ➃ | ➄ | ➅ |
|  | Middle income | ➀ | ➁ | ➂ | ➃ | ➄ | ➅ |
|  | Higher-income | ➀ | ➁ | ➂ | ➃ | ➄ | ➅ |
| Friends/ colleagues | Lower-income | ➀ | ➁ | ➂ | ➃ | ➄ | ➅ |
|  | Middle income | ➀ | ➁ | ➂ | ➃ | ➄ | ➅ |
|  | Higher-income | ➀ | ➁ | ➂ | ➃ | ➄ | ➅ |
| Neighbors | Lower-income | ➀ | ➁ | ➂ | ➃ | ➄ | ➅ |
|  | Middle income | ➀ | ➁ | ➂ | ➃ | ➄ | ➅ |
|  | Higher-income | ➀ | ➁ | ➂ | ➃ | ➄ | ➅ |

**C. PREFERENCE TO MIXED-INCOME NEIGHBORHOODS AND WILLINGNESS TO MOVE**

C1. Would you feel comfortable living in the following mixed-income neighborhoods?

Note: Each person represents a household, **a white person** is a household whose income level is similar to yours, and **a black person** is a household whose income level is **different from** yours.

| **Spatial distribution** | **Income levels** | **Strongly uncomfortable** | **Uncomfortable** | **Comfortable** | **Strongly comfortable** |
| --- | --- | --- | --- | --- | --- |
| 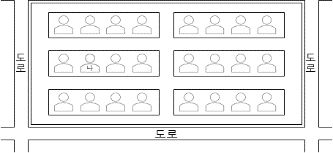 | Homogenous | ➀ | ➁ | ➂ | ➃ |
| 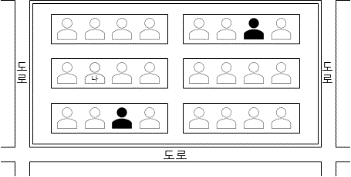 | Lower-income | ➀ | ➁ | ➂ | ➃ |
|  | Middle income | ➀ | ➁ | ➂ | ➃ |
|  | Higher-income | ➀ | ➁ | ➂ | ➃ |
| 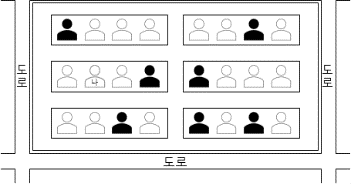 | Lower-income | ➀ | ➁ | ➂ | ➃ |
|  | Middle income | ➀ | ➁ | ➂ | ➃ |
|  | Higher-income | ➀ | ➁ | ➂ | ➃ |
| 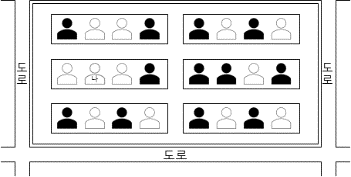 | Lower-income | ➀ | ➁ | ➂ | ➃ |
|  | Middle income | ➀ | ➁ | ➂ | ➃ |
|  | Higher-income | ➀ | ➁ | ➂ | ➃ |
| 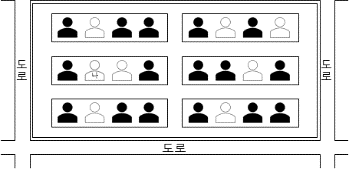 | Lower-income | ➀ | ➁ | ➂ | ➃ |
|  | Middle income | ➀ | ➁ | ➂ | ➃ |
|  | Higher-income | ➀ | ➁ | ➂ | ➃ |

C2. Would you be willing to move into the following mixed-income neighborhoods?

| **Spatial distribution** | **Income levels** | **Yes** | **No** |
| --- | --- | --- | --- |
| 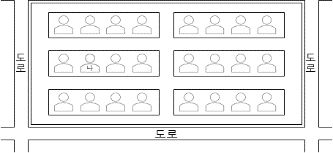 | Homogenous | ➀ | ➁ |
| 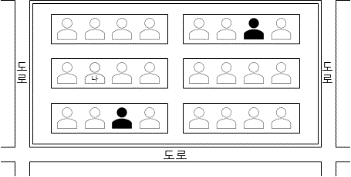 | Lower-income | ➀ | ➁ |
|  | Middle income | ➀ | ➁ |
|  | Higher-income | ➀ | ➁ |
| 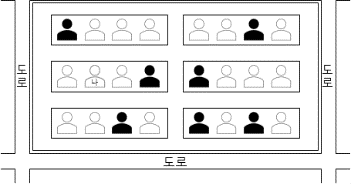 | Lower-income | ➀ | ➁ |
|  | Middle income | ➀ | ➁ |
|  | Higher-income | ➀ | ➁ |
| 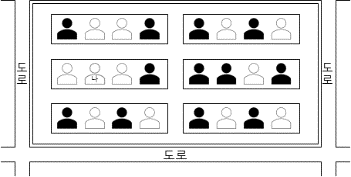 | Lower-income | ➀ | ➁ |
|  | Middle income | ➀ | ➁ |
|  | Higher-income | ➀ | ➁ |
| 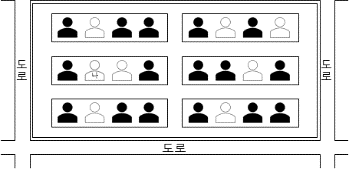 | Lower-income | ➀ | ➁ |
|  | Middle income | ➀ | ➁ |
|  | Higher-income | ➀ | ➁ |
