# Supplementary material for: Personal attitude or experience? Which factors influence residents’ acceptance of mixed-income communities?
Source: PLoS One. 2021 Apr 23;16(4):e0250511. doi: 10.1371/journal.pone.0250511 (PMC8064591; doi:10.1371/journal.pone.0250511)
Supplement: Attachment — Submitted filename: Response to reviewers_1st_revision_final.docx [file pone.0250511.s003.docx]

**Response to Reviewer’s Comments and Suggestions [PONE-D-20-38542]**

**We very much appreciate the thoughtful recommendations and comments of the reviewers and editor. Based on the feedback, we made revisions by clarifying the explanations and interpretation of the results. Also, we provided the survey questionnaires in Appendix as supporting information, and the correspondence between survey questionaries and variables is presented in Table 1. Our revisions were described under each comment of the reviewer.**

**Editor**

*1. Please ensure that your manuscript meets PLOS ONE's style requirements, including those for file naming. The PLOS ONE style templates can be found at*

**We tried to comply with all PLOS ONE’s style requirements, including the name of the files to submit.**

*2. Please provide a copy of the questionnaire used in the study, as a supplemental file.*

**Because the survey questionaries of this study are a part of the study about shared housing, only some parts of the original survey questionnaires were actually used for this study. Instead of providing the entire survey questionnaires which include less relevant items with this study, we provided the survey questionaries translated into English used for this study as supporting information in the Appendix.**

*3. We note that you have indicated that data from this study are available upon request. PLOS only allows data to be available upon request if there are legal or ethical restrictions on sharing data publicly. For more information on unacceptable data access restrictions, please see* [*http://journals.plos.org/plosone/s/data-availability#loc-unacceptable-data-access-restrictions*](http://journals.plos.org/plosone/s/data-availability#loc-unacceptable-data-access-restrictions)*.*

*In your revised cover letter, please address the following prompts:*

*a) If there are ethical or legal restrictions on sharing a de-identified data set, please explain them in detail (e.g., data contain potentially sensitive information, data are owned by a third-party organization, etc.) and who has imposed them (e.g., an ethics committee). Please also provide contact information for a data access committee, ethics committee, or other institutional body to which data requests may be sent.*

*b) If there are no restrictions, please upload the minimal anonymized data set necessary to replicate your study findings as either Supporting Information files or to a stable, public repository and provide us with the relevant URLs, DOIs, or accession numbers. For a list of acceptable repositories, please see* [*http://journals.plos.org/plosone/s/data-availability#loc-recommended-repositories*](http://journals.plos.org/plosone/s/data-availability#loc-recommended-repositories)*.*

*We will update your Data Availability statement on your behalf to reflect the information you provide.*

**There are no ethical or legal restrictions on sharing a de-identified data set. Thus, we uploaded the minimal anonymized data set. I provided the statement in the cover letter.**

*4. Thank you for stating the following in the Acknowledgments Section of your manuscript:*

*'This work was supported by the National Research Foundation of Korea (NRF) Grant funded by the Korean Government(MSIP) (No.NRF-2015R1A5A7037825).'*

*We note that you have provided funding information that is not currently declared in your Funding Statement. However, funding information should not appear in the Acknowledgments section or other areas of your manuscript. We will only publish funding information present in the Funding Statement section of the online submission form.*

*a. Please remove any funding-related text from the manuscript and let us know how you would like to update your Funding Statement. Currently, your Funding Statement reads as follows:*

*'The funders had no role in study design, data collection and analysis, decision to publish, or preparation of the manuscript.'*

*b. Please include your amended statements within your cover letter; we will change the online submission form on your behalf.*

**As you suggested, we removed the funding information in the Acknowledgement. As I initially noted by the funder, the National Research Foundation of Korea, has no specific role in this research, except providing the funding. As this study is the outcome of the funded research, the funding information should be provided. I would add these two sentences.**

**'This work was supported by the National Research Foundation of Korea (NRF) Grant funded by the Korean Government(MSIP) (No.NRF-2015R1A5A7037825).'**

**'The funders had no role in study design, data collection and analysis, decision to publish, or preparation of the manuscript.'**

**Reviewer 1**

*Well-written; introduction lays out a strong argument of why this study is important. Unique perspective to focus on the consumer in regards to mixed-income communities.*

*p.3 1st paragraph: “Yonker” should be “Yonkers”*

**We are sorry for this mistake. We revised it (See Line 56).**

*p.5 last paragraph, “as seen” should be added before “in some stigmatized French neighborhoods”*

**As suggested, we added “as seen” to clarify the meaning of the sentence (See Line 166)**

*p.6 “The processes of economic reform and institutional transition under neo-liberalism have affected residents’ ideology and value judgment, leading to a change in residents’ preferences and support for inclusive housing policies in Chinese cities [35]. Accordingly, more residents have come to accept economic individualism, which is possibly an emerging challenge for social mix [35].” – this seems contradictory?*

**We are sorry for the confusion. To deliver the findings of the cited study more clearly, we rewrote the sentences (See Lines 193-200).**

***The processes of economic reform and the institutional transition could affect residents’ ideology and value judgment, leading to a change in residents’ preferences to inclusive housing policies [35]. Specifically, compared to the elderly having a strong belief in social equality, the younger generation who is likely to support economic individualism, tends to have lower preferences for inclusionary housing in Jiangsu Province, China [35].***

*What was response rate of survey? If low, this should be noted as a limitation*

**The response rate of the survey is not available because the MatriX Corporation conducted the survey with their online-panels and the company does not provide specific information about the response rate. Instead, we asked the MatriX Corporation to complete the survey to meet our stratified sampling requirements by region and age. As we know, the MatriX Corporation has about 400,000 online survey panels and some of them participated in our survey. Relevant information is provided in Lines 257-259.**

*Helpful to explain the similarities and differences of the 7 metropolitan areas chosen and why these 7 were chosen. Any city differences in results?*

**In Korea, the seven cities are the only metropolitan municipalities. All other cities are local (lower level) municipalities. We provided a further explanation about these seven cities (See Lines 256-257).**

**Our research does not focus on the differences by regions thus we included them as a control variable (regional dummy variables). Overall, there are only a few differences between cities as shown in Table 3. Respondents in Incheon and Busan showed statistically different results. The similarities and differences between cities could be future research topics. Thus, we only included the results without further interpretations in Table 3.**

*An appendix with a copy of the survey would be helpful for readers to review.*

**As suggested, the survey questionnaires translated into English are provided in the Appendix.**

*Table 1: Political Propensity shows max at 1, but this variable shows progressive as being 2. That would lead me to believe that max is 2, not 1?*

**The political propensity consists of two dummy variables. Thus, the maximum value of each dummy variable is 1.**

*Top of page 10: “wights” should be “weights”.*

**We are sorry for this typo. We correct it (See Line 348)**

*Top of page 11: why were 10 responses excluded?*

**In the original manuscript, 10 responses were excluded due to the lack of data availability of neighborhood quality (median housing price). In this revision, we check the data availability again and finally found that the median housing price information for 6 responses is available. Therefore, we updated the data sets and conducted the analysis, again. Overall, the results are almost the same. We provided the reasons why 4 responses are not used due to the lack of data availability (See Lines 354-357).**

*Table 1, Figure, 2, Table 2, and Figure 3 should be placed after the first paragraph where it is referenced, not a few paragraphs below*

**As suggested, we changed the position of these tables and figures after the first paragraph where it is referenced.**

*Results seem to confirm much of the existing literature and this should be noted in the discussion as well as what existing literature it confirms.*

**As suggested, we noted that our results are consistent with previous studies and confirm existing literature in the discussion part. Also, if three is a difference between our findings and the findings of existing literature, we added further explanations. (See Lines 412; 418-419; 461-464).**

**Reviewer 2**

*I did not see a data set that you had submitted, but I might have missed it. Please provide that.*

**As suggested, we provided the data sets we used as a supplementary file.**

*On page 2, your reference to “the actual field of communities” seems a little awkward. Substituting a phrase in there such as “urban planning” or “community development” might be less awkward.*

**As suggested, we revised it to “community development” (See Line 48)**

*On page 3, I am wondering if the “Yonker” program should actually be “Yonkers”.*

**We are sorry for this mistake. We correct it (See Line 56)**

*On page 6, you mention “social contact theory” and am wondering if you might have meant “social contract theory”.*

**To clarify the theory we mentioned, we use “intergroup contact theory” instead of using “social contact theory”. The theory is based on Pettigrew (1998). We added the reference and short explanations about the theory (See Lines 205-207).**

*In the survey sampling design, it is not clear how the populations were chosen. Was there a database of mixed income properties? How many properties are we talking about here?*

**The survey was conducted to understand the perceptions of the general public instead of understanding the perceptions of the residents in mixed-income communities. The stratified sampling by regions and ages is based on the Population and Housing Census. We provided explanations. (See Lines 251-254).**

*In Figure 1, is this your own way of operationalizing degree of mix? Panels 4 and 5 look pretty much the same to me.*

**Based on Farley et al., (1997), we developed our own operational descriptions for five different levels of mixed-income communities. In particular, we intended that the respondent is a majority in the slightly mixed and moderately mixed neighborhoods while the respondent is a minority in the extremely mixed neighborhoods (See Lines 275-279).**

*I like that you used the income diversity index…this seems like the proper way to determine some form of mixed. Having said that, I think adding a sentence or two about the Simpson index methodology might be appropriate here as well.*

**As suggested, we added a short description of the conceptual meaning of the Simpson index. We do not provide a specific equation we used because it is easily found in many other studies. Instead, we provided a reference that used the Simpson index for income diversity study (Kim, 2016 [25]). (See Lines 305-307).**

*On page 9, you mentioned a foreigner rate in an address registration data base. I am not familiar with this concept. Also, if the focus is on degree of mix by income level, what does foreign-born have to do with anything?*

**The foreigner rate was introduced as a variable to represent a diverse neighborhood environment in Korea. Because Korea is a homogeneous country in terms of race and ethnicity, the foreigner rate in a neighborhood could be a proxy of social diversity. We assumed that if people live in neighborhoods with higher foreigner rate, they are more likely to have more opportunities to communicate with foreigners, which represent the experience living in diverse communities. We added a more specific explanation and reasoning using the foreigner rate. (See Lines 309-315).**

*Also, if you could convert the currency into dollars, that would be helpful to US readers to understand perspective.*

**We agree with your suggestion. But, the readers are not limited to scholars in the US. Therefore, we decided to provide the currency information in the note of Table 2. (*1 US $ is equivalent to 1,100 KRW*). We hope you agree to this revision. (See Line 322).**

*It might not be a bad idea to include the survey instrument as an appendix. It seems like the manuscript has enough room for it to be included.*

**As suggested, the survey questionnaires translated into English are provided in the Appendix.**

*On page 13, I do not think that the interpretation of the coefficient for community attachment is correct. Instead of .81 times lower acceptance, it is technically .81 times of average acceptance for those with low scores for community attachment. Perhaps it is better to say 19% lower?*

*You make the same interpretation issues with the other variables as well. For example, with the statement “Lower-income individuals who often meet higher-income people demonstrated about 1.3 times higher acceptance than those who rarely interact with higher-income people”, it should actually be “30% higher acceptance”. The way it reads now, it seems like it almost be 130% higher. Please go through all of these coefficient interpretations.*

**As suggested, we revised the interpretations of the ordinal regression models. Because the exact interpretations of estimated odds ratios are different depending on the types of independent variables (continuous variables or dummy variables), we revised and clarified the interpretations considering the types of independent variables. When showing the odds ratios are needed to compare the results, we kept using the term “OO times” with an expression of “the odds of~”. We believe that our revisions could provide more exact and clear interpretations of the results. (See Lines 410; 420-421; 435-436; 444-445; 457-459; 468-470).**
